# Growing season carries stronger contributions to *albedo* dynamics on the Tibetan plateau

**DOI:** 10.1371/journal.pone.0180559

**Published:** 2017-09-08

**Authors:** Li Tian, Jiquan Chen, Yangjian Zhang

**Affiliations:** 1 Qianyanzhou Ecological Research Station, Key Laboratory of Ecosystem Network Observation and Modeling, Institute of Geographic Sciences and Natural Resources Research, Chinese Academy of Sciences, Beijing, China; 2 Lhasa station, Key Laboratory of Ecosystem Network Observation and Modelling, Institute of Geographic Sciences and Natural Resources Research, Chinese Academy of Sciences, Beijing, China; 3 CGCEO and Department of Geography, Environment, and Spatial Sciences, Michigan State University, East Lansing, Michigan, United States of America; Pacific Northwest National Laboratory, UNITED STATES

## Abstract

The Tibetan Plateau has experienced higher-than-global-average climate warming in recent decades, resulting in many significant changes in ecosystem structure and function. Among them is *albedo*, which bridges the causes and consequences of land surface processes and climate. The plateau is covered by snow/ice and vegetation in the non-growing season (nGS) and growing season (GS), respectively. Based on the MODIS products, we investigated snow/ice cover and vegetation greenness in relation to the spatiotemporal changes of *albedo* on the Tibetan Plateau from 2000 through 2013. A synchronous relationship was found between the change in GSNDVI and GSalbedo over time and across the Tibetan landscapes. We found that the annual average *albedo* had a decreasing trend, but that the *albedo* had slightly increased during the nGS and decreased during the GS. Across the landscapes, the nGS*albedo* fluctuated in a synchronous pattern with snow/ice cover. Temporally, monthly snow/ice coverage also had a high correspondence with *albedo*, except in April and October. We detected clear dependencies of *albedo* on elevation. With the rise in altitude, the nGS*albedo* decreased below 4000 m, but increased for elevations of 4500–5500 m. Above 5500 m, the nGS*albedo* decreased, which was in accordance with the decreased amount of snow/ice coverage and the increased soil moisture on the plateau. More importantly, the decreasing *albedo* in the most recent decade appeared to be caused primarily by lowered growing season *albedo*.

## Introduction

The magnitude of global warming in recent decades on the Tibetan Plateau—the largest plateau in the world—has surpassed the average of the northern hemisphere. The warming is causing a series of changes in terrestrial surface properties, which in turn feed back to regional and global climate [1]. Among the various land surface properties, *albedo* is one of the most critical variables because it bridges land surface processes (e.g., land use, vegetation dynamics) and climate [2–4]. Within the climate system, *albedo* determines surface radiation balance and affects surface temperature. For the ecological systems, *albedo* affects energy balance (including evapotranspiration) through regulating the microclimatic conditions of plant canopies and their absorption of solar radiation. Consequently, *albedo* has been substantially studied for its role in global change science (e.g., [5–7]), including its role in the development of climate change scenarios. The magnitude and change in *albedo* are tightly related to land cover type and other surface properties such as snow/ice cover [8], vegetation [4, 9–10], landform [11], and soil moisture [12]. Globally, *albedo* is reportedly decreasing in the Arctic Circle due to shrunken snow/ice cover [13], in boreal biomes [14], and in other regions. At high altitudes in mountainous areas, dense vegetation appears to reduce radiative forcing [4,8,15–16].

*Albedo* is a key surface characteristic mediating the surface’s energy balance. The Tibetan plateau covers 2.57 million km^2^ and serves not only as a major water source for South and East Asia, but also as the “heat pump” that regulates the Asian climate. A shifted *albedo* regime on the Plateau would not only modify the “heat pump” phenomenon for the East Asian monsoon, but would also amplify regional warming through permafrost thawing [17]. Both of the above causal relationships would change summer precipitation in East Asia [18].

Climatic and land use changes can directly increase/decrease land surface *albedo*. On the Plateau, snow/ice is the dominant land cover type during the non-growing season (nGS; October–April), while vegetation (mostly grasslands) dominates the growing season (GS; May–September) [19]. Substantial efforts have been made to quantify the dynamics of vegetation on the plateau in correspondence to the rapid climatic change [20–21], as well as the ecological consequences [22]. The causes and consequences of the spatiotemporal changes in *albedo* have also been investigated [20–21], with clear evidence showing the direct, significant influences from the change in snow/ice [21, 23] and vegetation. However, no scientific effort has been made to understand if these changes are equal between the GS and the nGS. More importantly, the quantitative contributions of changes in snow/ice cover and vegetation to the spatiotemporal dynamics of *albedo* across the heterogeneous landscapes on the plateau are yet to be explored.

In this study, we aimed to investigate the independent effects of vegetation and snow/ice on *albedo* changes on the Tibetan Plateau. Specifically, our objectives were to: (1) explore the spatial and temporal changes of *albedo* for its annual, GS, and nGS values on the Tibetan Plateau; and (2) investigate the potential driving forces propelling *albedo* dynamics. This study advances the understanding of the driving mechanisms behind *albedo*’s spatiotemporal dynamics on the plateau and improves the accuracy of predictions by including the feedback effects of land cover changes to the change in global climate.

## Materials and methods

### Study site

**Fig 1:**
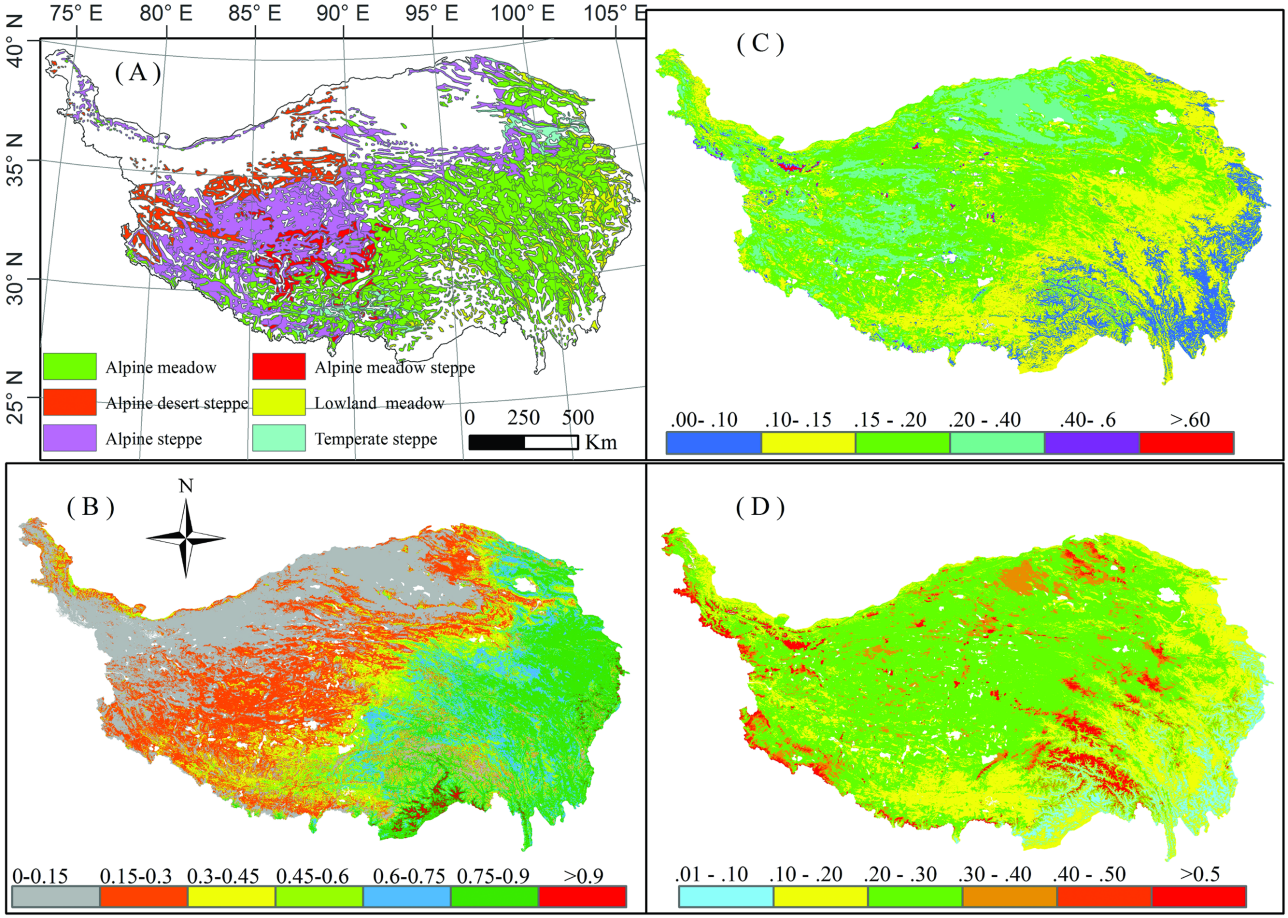
Study area and the distribution of vegetation, NDVI, and *albedo* during 2000–2013 on the Tibetan Plateau: (A) vegetation, (B) average GS_NDVI_ (May–September), (C) average GS*albedo* (May–September), and (D) average nGS*albedo* (January–April and October–December).

The Tibetan Plateau extends from subtropical to mid-latitude regions, spanning 13° latitude and 25° longitude (26.5–39.5°N, 78.3–103°E) (Fig 1A). The mean temperature of the coldest and the warmest month is approximately -10°C and 10°C, respectively [24]. Temperature and precipitation have distinct decreasing gradients from the southeast to the northwest. The average base elevation is ~4000 m.

Alpine grassland is the dominant vegetation type that covers ~70% of the plateau, based on the spatial data from the Data Center of Resources and Environmental Sciences [25] (Fig 1A). Along the climatic gradient from the southeast to the northwest, six land cover types stretch across the plateau: alpine meadow (47.5%), alpine steppe (30.98%), alpine desert steppe (7.41%), alpine meadow steppe (4.21%), low land meadow (6.74%), and temperate steppe (3.61%) (Fig 1A). Sparse vegetation grows in the arid northwestern mountains, with a very low Normalized Difference Vegetation Index (NDVI) of <0.1. A relatively small area of evergreen forest can be found in the southeast where it is warm and wet, with an NDVI of >0.8. We excluded forests in this study due to the contrasting and complex relationships between forests and *albedo* [26].

### Data sources

We used the baseline dataset of the Moderate Resolution Imaging Spectroradiometer (MODIS) onboard NASA’s Earth Observing System’s satellite Terra (including snow/ice cover, NDVI and *albedo* products) to quantify the spatiotemporal changes of *albedo* on the plateau. For the GS, the GSNDVI and GS*albedo* data were used; whereas for the nGS, nGS*albedo* and non-growing season snow/ice cover (non-GSSC) were used. The retrieved data was further improved by filtering out noises that were caused by cloud contamination and topographic differences using subset tools (http://daac.ornl.gov/MODIS/modis.shtml).

The MODIS Terra NDVI products have been extensively validated on the plateau. The MOD13Q1 (2000–2013) is a 16-day composite product with a 250 m spatial resolution. For the GS, the dataset included a total of 10 NDVI images produced between DOY (Day of Year) 129 and DOY 273 (May 8–September 30). We calculated the maximum monthly values from the two adjacent NDVI images by month and by pixel. For example, the data for May was extracted as the maximum value between DOY 129 and DOY 145 for each pixel (Eqs 1 and 2).

The MODIS *albedo* products have also been validated extensively [27] and applied on the Plateau (e.g., serving as a benchmark for evaluating other satellite *albedo* products). We used the MODIS shortwave product of the collection5 (MOD43A3), which had been generated every eight days at 500 m spatial resolution since early 2000. The dataset was calculated by comparing black-sky *albedo* for direct values to white-sky *albedo* (WSA) for isotropic diffuse radiation at local solar noon [28]. Because our objective was to explore the *albedo* dynamics as well as the regulatory mechanisms from the vegetation and snow/ice cover, the WSA products reflecting the true condition of the surface land cover were used in this study [29]. The annual products included a total of 48 images between DOY 001 and DOY 361 (January 1–December 30).

For the GS, we used the 20 *albedo* images produced between DOY 121 and 273 (May 1–September 30) as the GS*albedo*. We calculated the minimum monthly values and the annual means from the four adjacent *albedo* images by month and by pixel for the GS (Eqs 3 and 4). For the nGS, we used the remaining images for DOY 001–113 and DOY 281–361 (January 1–April 30 and October 1–December 30, respectively) and calculated the mean monthly values from the four adjacent *albedo* images by month and by pixel for 2000–2013 (Eq 5).

Among the FY-2E VISSR, MODIS, and IMS snow/ice products, the MODIS snow/ice cover data presented the highest overall accuracy, especially in mitigating cloud cover effects and processing grassland types [30]. Here, we used the Level 3 data of snow/ice data products (MOD10A2), which was the 8-day composite at 500 m resolution. For this data set, we labeled each 500 m pixel as barren land, snow/ice, cloud obscured, water, etc., to extract the pixels with snow/ice from each image and compile the cell as non-GSSC data. At the annual scale, a total of 26 snow/ice cover images were produced during DOY 001–113 and DOY 281–361 (January 1–April 30 and October 1–December 30, respectively) to count the mean number from the four adjacent snow/ice cover images by month and by pixel (Eq 6).

Soil moisture is a critical variable reflecting the changes in water and energy balance at the land’s surface. It is also one of the most important input variables in popular meteorology, agronomy, ecology, and hydrology models. In this study, soil moisture estimates were generated using a change detection algorithm from scatterometers (SCAT) onboard the European Remote Sensing satellites (ERS-1/2) from 1991 [31], with a 0.25° regular grid for daily averages. The satellites coarse spatial resolution results in spatial heterogeneity in one pixel, lending many uncertainties to the parameter retrieval and creating challenges for product validation. Here, we only used it to assist in explaining the correlation between nGSSC and nGSAlbedo. For this data set, at the annual scale, a total of per day images were produced during January 1–April 30 and October 1–December 30, respectively. These per day images were utilized to to calculate the average monthly value from the per day images by pixel. At the annual scale, the average annual value was counted by month and by pixel (Eq 7).

### Quantitative metrics and analysis

The annual minimum GS*albedo* occurred simultaneously with the annual maximum GSNDVI. A correlation analysis was performed to explore the relationship between vegetation greenness and *albedo* by matching the annual minimum/maximum GS*albedo*/GSNDVI and the annual average GS*albedo*/GSNDVI for the GS, respectively. The annual GSNDVI_max_ and GSNDVI_mean_ were calculated as:

$${\text{GSNDVI}}_{max}=\text{Max }({\text{NDVI}}_{max,i})$$ (1)

$${\text{GSNDVI}}_{mean}=\frac{1}{5}\times \sum_{i=1}^{5}{\text{NDVI}}_{max,i}$$ (2)

where GSNDVI_max_ and GSNDVI_mean_ are the annual maximum NDVI and the annual average value in the GS, respectively. NDVI_*max*,*i*_ is the maximum NDVI of the month for the GS, and *i* represents the months (e.g., May, June, July, August, and September) in a year.

The annual GS*albedo*_min_ and the GS*albedo*_mean_ were calculated for each pixel as:

$$\text{GS}albedo_{min}=\text{Min (}albedo_{min,i})$$ (3)

$$\text{GS}albedo_{mean}=\frac{1}{5}\times \sum_{i=1}^{5}albedo_{min,i}$$ (4)

where GS*albedo*_*min*_ is the annual minimum *albedo* in the GS, GS*albedo*_*mean*_ is the annual average value of the GS, *Albedo*_*min*,*i*_ is the minimum *albedo* of each month for the GS, and *i* represents the month of a year. We matched the annual average nGS*albedo* with the annual mean number of non-GSSC, which was calculated as:

$$\text{nGS}albedo_{mean}=\frac{1}{7}\times \sum_{m=1}^{7}albedo_{mean,m}$$ (5)

$${\text{nGSSC}}_{mean}=\frac{1}{7}\times \sum_{m=1}^{7}{\text{GSSC}}_{mean,m}$$ (6)

where nGS*albedo*_*mean*_ and nGSSC_*mean*_ are the annual average *albedo* and mean number of snow/ice cover fraction in the nGS, respectively. The *albedo*_*mean*,*m*_ and GSSC_*mean*,*m*_ are the mean *albedo* and snow/ice-cover of each month during the nGS, where m represents the months (e.g. January, February, March, April, October, November, December). We produced a spatially-continuous database using regression models based on the time series of the original field at each grid point. The snow/ice cover data was in the format of 0 (non-presence) and 1 (presence). We calculated the percentage of presence for specified periods.

The microwave soil moisture data has a daily resolution. First, the annual nGSsoil moisture_*mean*_ was calculated for each pixel as:

$${\text{nGSsoil moisture}}_{mean}=\frac{1}{7}\times \sum_{m=1}^{7}{\text{GSsoil moisture}}_{mean,m}$$ (7)

To explore the altitude-dependent relationships of GS*albedo* and GSNDVI, or nGS*albedo* and non-GSSC, we split the plateau into 500 m elevation bands and defined the trend slopes of the two datasets as the rate of change. Finally, we explored the causal effects on *albedo* through regressing vegetation greenness in the GS and the snow/ice cover in the nGS against *albedo* for the corresponding periods.

## Results and discussion

### The spatiotemporal changes of *albedo*

From the southeast to the northwest of the Tibetan Plateau, there exists a mix of forests, meadows, steppes, and desert steppes, with decreasing vegetation greenness along this gradient (Fig 1B). The highest and the lowest GS_NDVI_ were found at the alpine meadow (~0.8) and the alpine desert steppe (~0.2) (Fig 1A and 1B). The GS*albedo* increased from ~0.1 to 0.4 from the southeast to the northwest (Fig 1C), while the nGS*albedo* varied between 0.1 and 0.3 along the gradient from the southeast to the northwest. Pixels with nGS*albedo* values of >0.3 were found in high altitude areas where vegetation dotted the bare landscapes (Fig 1D). The average GS*albedo* for alpine meadow, alpine steppe, alpine desert steppe, alpine meadow steppe, lowland meadow, and temperate steppe was 0.17, 0.22, 0.22, 0.21, 0.14, and 0.18, respectively. The average nGS*albedo* for alpine meadow, alpine steppe, alpine desert steppe, alpine meadow steppe, lowland meadow, and temperate steppe was 0.22, 0.25, 0.26, 0.23, 0.15, and 0.18, respectively. The *albedo* in nGS was significantly higher than that in GS for all cover types.

**Fig 2:**
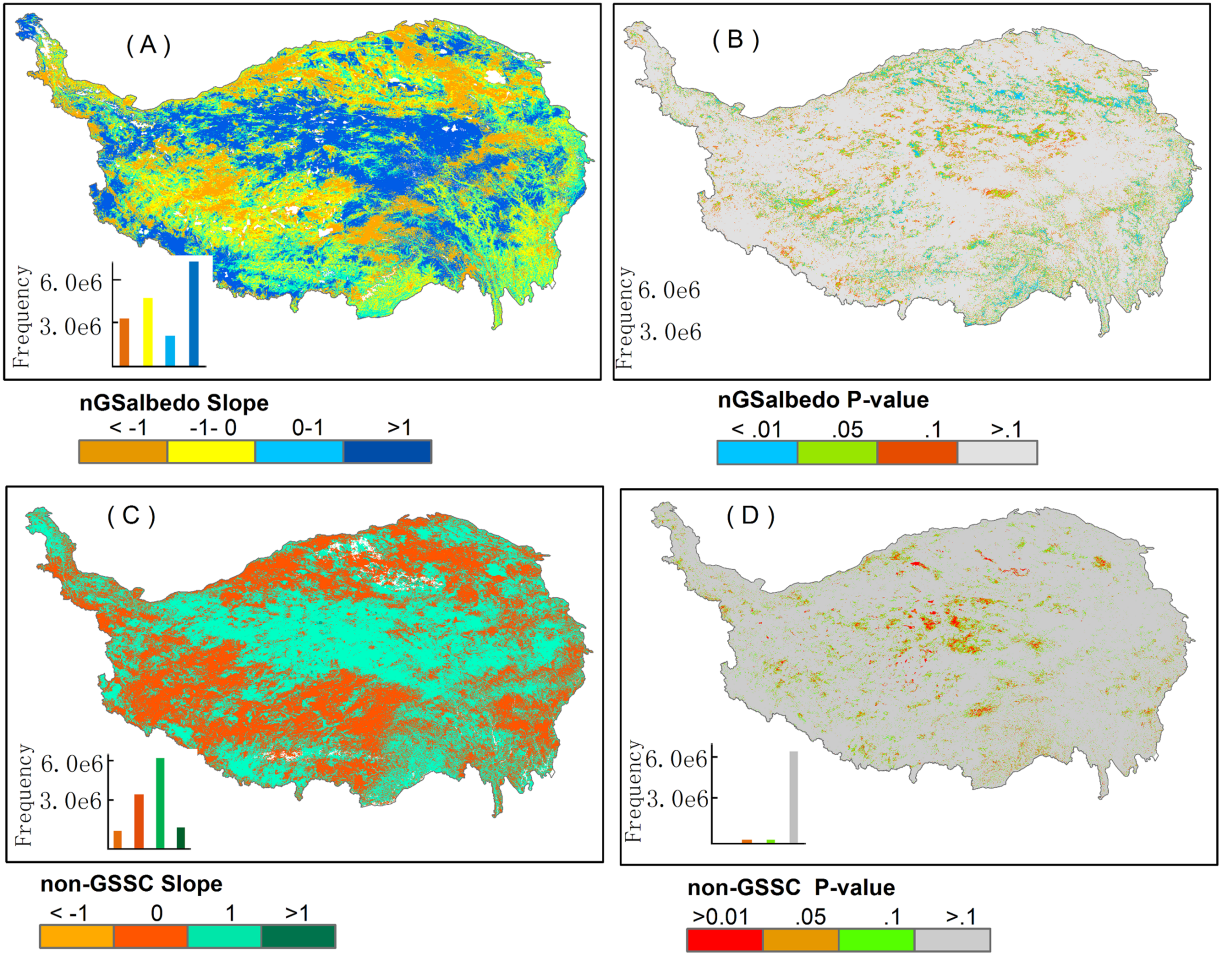
Spatial patterns of the changing rates (i.e., slope of the regression line) and the spatial changes in the corresponding *P* values from 2000 through 2013: (A) nGS*albedo*, (C) non-GSSC, and (B) and (D) are labels at four significance levels. The slopes of nGSalbedo and non-GSSC change trends are divided into several levels representing their change magnitudes. The P values (B) and (D) are divided into four significance levels: P<0.01, 0.01<P<0.05, 0.05<P<0.1, P>0.1.

The temporal change of nGS*albedo* exhibited clear spatial variation across the Plateau (Fig 2A), with more pronounced values in the northern and southeastern plateau, low values in the northeast and the southwest (Fig 2A), and high values in the northern plateau (Fig 2B). Compared to the temporal trend of GS*albedo* reported in our previous study [32], the changing trend of nGS*albedo* appeared less obvious, with a larger proportion of areas that had witnessed a decreasing trend in GS*albedo* but not in nGS*albedo*.

**Fig 3:**
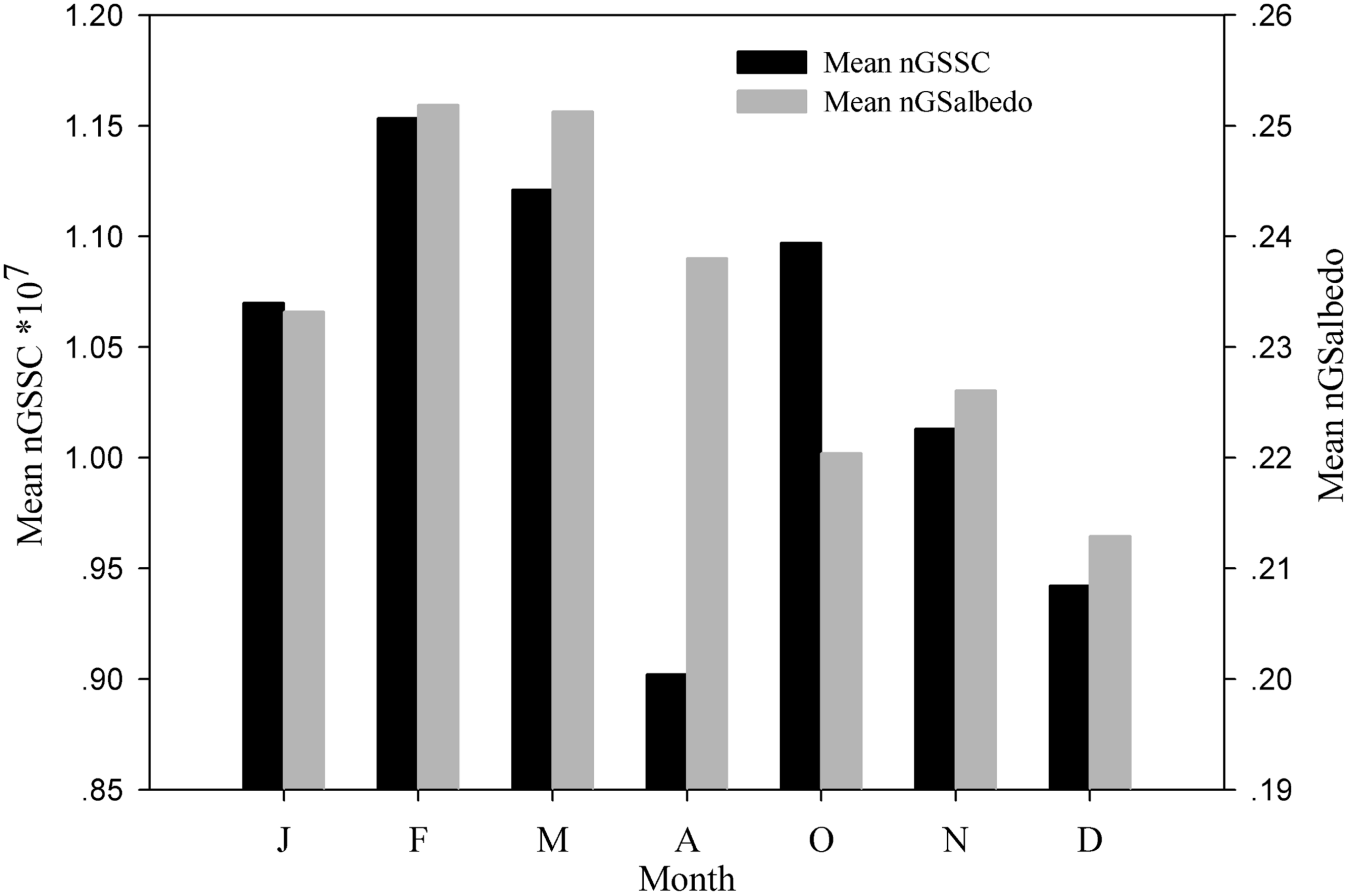
Average changes in land areas of snow/ice cover and nGSalbedo by month in nGS on the Tibetan Plateau during 2000–2013.

The nGS*albedo* fluctuated in a synchronous pattern with snow/ice cover. Spatially, in areas where snow/ice expanded, nGS*albedo* increased (Fig 2A and 2C). Over time, monthly snow/ice coverage also followed a high correspondence with *albedo*, except in April and October (Fig 3). In April, the snow/ice started to melt due to the rising temperature, expanding the wet surfaces and encouraging vegetation to bud [14]. In October, snow/ice accumulation did not appear coupled with changes in *albedo*. The reason for this could be that residuals from withered vegetation in autumn slowed down the increasing rate of *albedo*.

Seasonally, the maximum nGS*albedo* occurred in early spring (>0.25) (February and March) and the minimum nGS*albedo* occurred in winter (0.213) (December). The highest non-GSSC cover was found in early spring (February and March) (>1.18×10^7^ pixel), with a sudden drop in April (Fig 3). By late autumn (October), there were 1.197×10^7^ pixels before it decreased to 1.043×10^7^ in November, 0.952×10^7^ in December, and 1.074×10^7^ in January. Interestingly, we found that snow/ice cover was higher in late autumn (October) than that of all winter, regardless of low *albedo* in November and December. In addition, snow/ice cover was the lowest in April, with a high *albedo*. Finally, it appeared that the minimum *albedo* occurred in December (i.e., the coldest month of a year), which would likely produce indirect consequences on the East Asian jet stream (EAJT) during the winters when snow/ice is transferred to the adjacent valleys [33].

### Altitude dependency of snow/ice effects on *albedo*?

**Fig 4:**
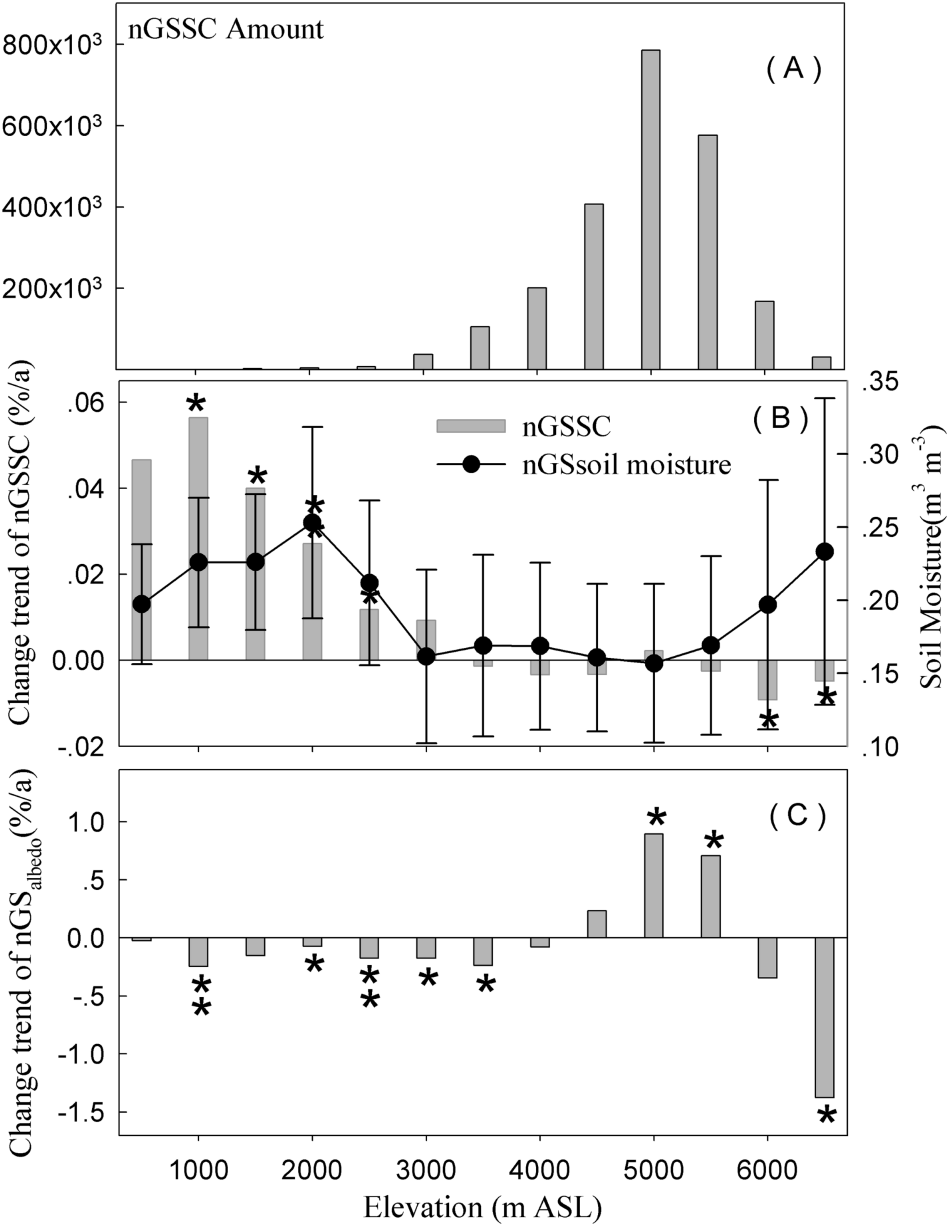
The changing rate of non-GSSC and nGS*albedo* by elevation for the grassland biome on the Tibetan Plateau, where * and ** indicate the statistical significance level at 95% and 99%, respectively.

We detected clear dependencies of *albedo* on elevation (Fig 4A). Below 3000 m, the non-GSSC increased from 2000 to 2013, but weakened in increments as elevation increased (Fig 4B). Between 3000 m and 6000 m, the non-GSSC decreased (Fig 4B), except at 5000–5500 m. Above 6000 m, where snow/ice persisted year-round, the non-GSSC decreased during 2000–2013 (Fig 4B). In synch with the expanding non-GSSC, however, the nGS*albedo* decreased below 3000 m (Fig 4C) and was significantly correlated with the amount of snow/ice. This was likely due to: (1) decreased snow cover at this elevation caused by the East Asian jet stream (EAJT) in the winter (Fig 4B); (2) the fact that the small amount of land area below 3000 m is only found in northeastern Tibet near the deserts, where snow/ice is primarily found in ravines running in a NW-SE direction; and (3) the high soil moisture below 2000 m (Fig 4B). Between 2000 m and 3000 m, the soil moisture appeared low, which coincided with high non-GSSC and the low nGS*albedo*. Between 3000 m and 5500 m the soil moisture was even lower, and the change of non-GSSC matched well with the nGS*albedo*. For the elevation of 4500 m–5500 m, the *albedo* showed an increasing trend (Fig 4C), especially for non-GSSC. Above 5500 m, the *albedo* decreased again, which was in line with the shrunken snow/ice coverage and elevated soil moisture (Fig 4B and 4C).

At high elevations (≥6000 m), both nGS*albedo* and non-GSSC decreased during the study period. The decreased snow cover at high elevations was consistent with Gardner *et al*., [34] who used satellite data to observe a steady reduction in snow cover in the Himalayan mountain regions. Essentially, the changes in the cryosphere are accompanied by a documented peak in temperature over Tibet, which is altitude dependent [35–37]; the decreased *albedo* was due to the shrinking snow/ice cover. Similar trends have also been reported in other regions [2,35, 38– 41]. For example, Scherrer *et al*. [42] found that the spring daily mean temperature in the Swiss Alps was 0.4°C higher in areas without snow/ice cover compared to those without snow/ice cover for 1961–2012.

**Fig 5:**
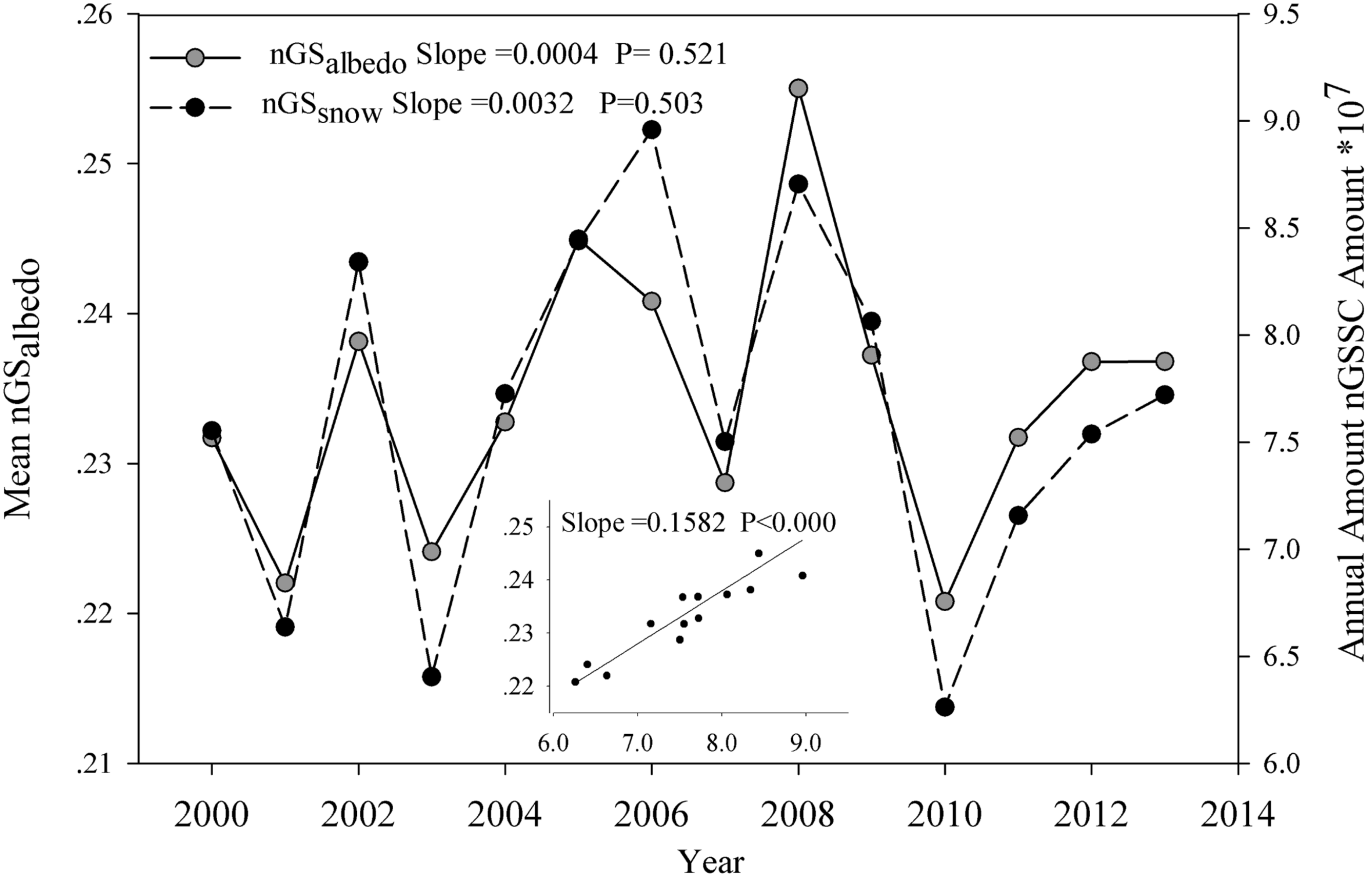
The changes in mean nGS*albedo* and the annual amount of non-GSSC in the grassland biome by year on the Tibetan Plateau.

The total snow/ice covered area in the nGS exhibited an insignificant changing trend during 2000–2013 (P>0.01), but with a wide range between 6.26×10^7^ and 8.96×10^7^ pixels (Fig 5). The annual average nGS*albedo* showed no obvious trends for 2000–2013, but a striking inter-annual fluctuation. The trajectory of non-GSSC coverage matched closely with that of the nGS*albedo* during 2000–2013 (slope = 0.158; *p*<0.001).

Over time, the changes in GS*albedo* appeared to be elevation dependent, which coincided with the elevation-dependent changes in climate across the plateau [43–44]. Temperature showed an increasing rate in the lower elevation zones during the GS. As a result, the contribution of vegetation greenness to *albedo* dynamics was enhanced from the foothills to 3500 m, with high R^2^ values [32].

**Fig 6:**
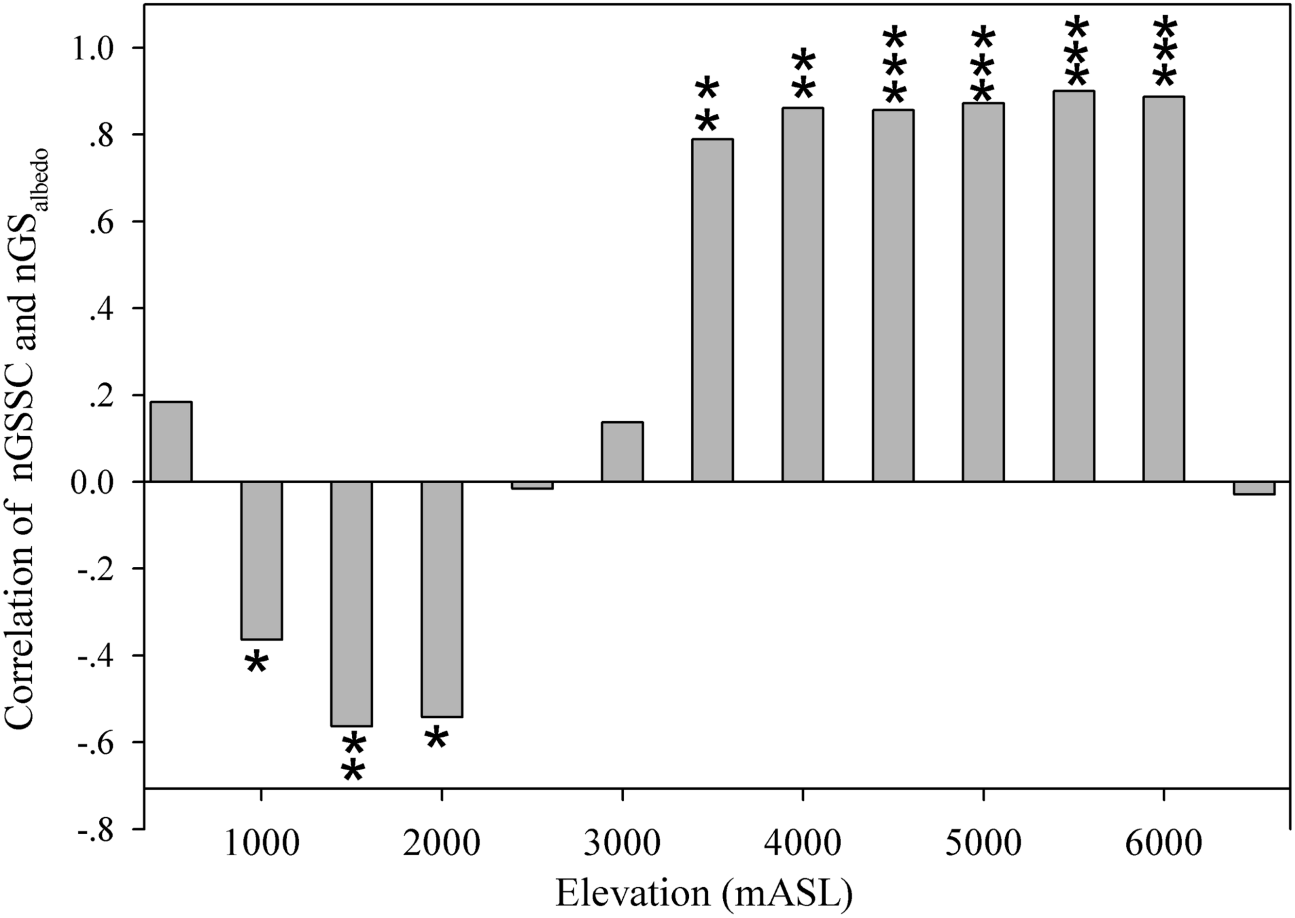
The elevation-dependent correlations between the mean non-GSSC and nGS*albedo*, where *, **, and *** indicate the statistical significance level at 95%, 99%, and >99%, respectively.

The correlation between nGS*albedo* and non-GSSC coverage varied among the altitudinal bins (Fig 6). Below 2000 m, the relation between snow/ice coverage and *albedo* was less clear in contrast with areas between 3000 m and 6000 m, where the relationship between *albedo* and snow/ice coverage was highly clear. Over 5500 m, the snow/ice coverage decreased and the soil moisture increased, making the relationship between *albedo* and snow/ice coverage ambiguous again (Figs 4 and 6).

From 3500 m to 5000 m, the contribution of vegetation greenness to the change in *albedo* appeared to weaken as vegetation became more sparse [32], while other factors (e.g., soil moisture) may have played stronger roles in influencing *albedo* and its dynamics. Above 3500 m, the effects of year-around snow/ice coverage emerged. Over 5500 m, vegetation cover was low and patchy (NDVI = 0.15), resulting in minor vegetation effects on *albedo*—even in the GS.

### Dynamics of annual *albedo*

**Fig 7:**
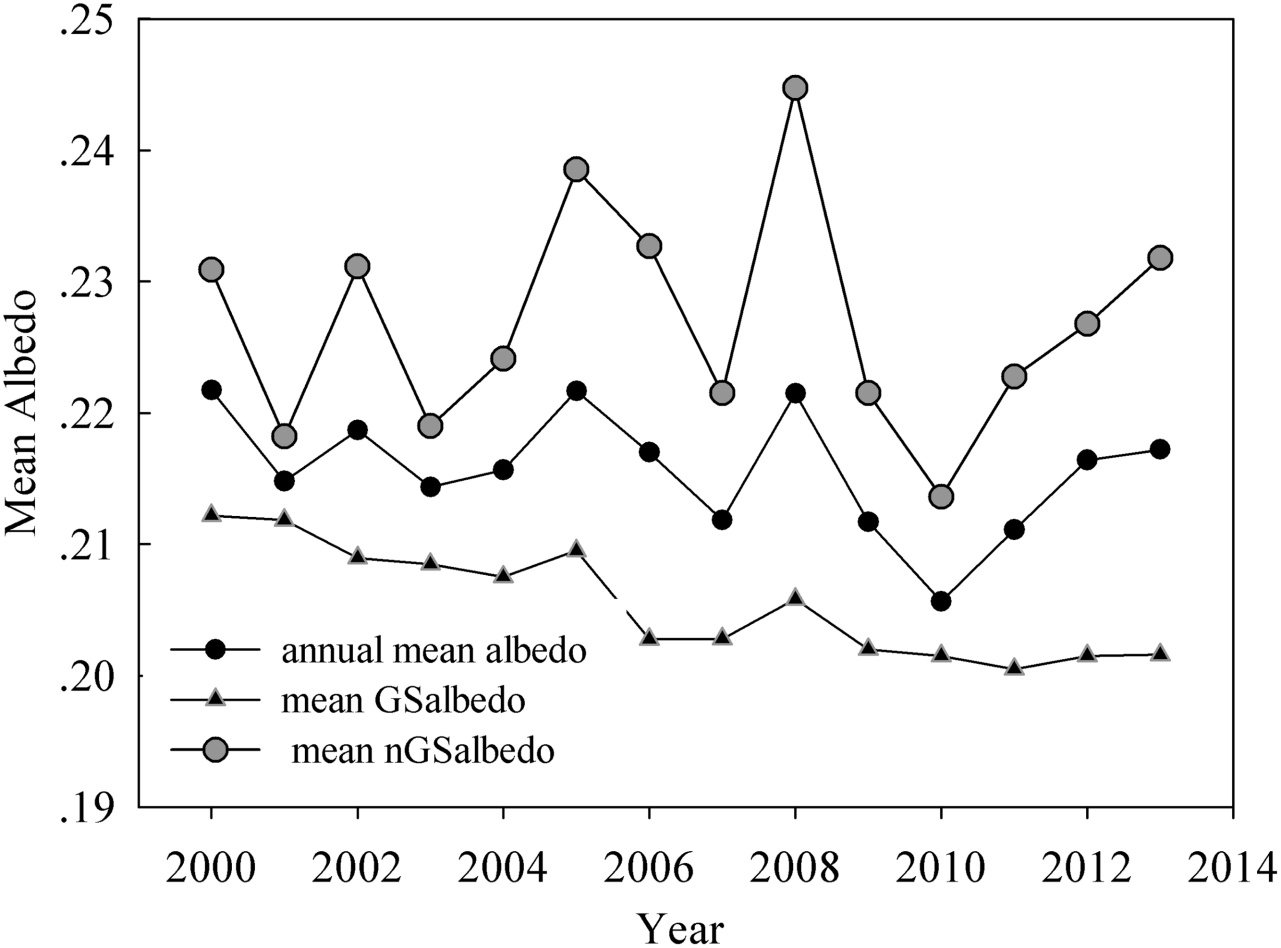
Changes in annual mean *albedo*, mean nGS*albedo* and mean GS*albedo* from 2000 through 2013 on the Tibetan Plateau.

The annual *albedo* continuously decreased during the study period (Fig 7), with nGS*albedo* and GS*albedo* following a slightly increasing (Slope = 0.0002, *P* = 0.742) and a significant decreasing (Slope = -0.0005, *P* <0.01) trend, respectively. The change in annual *albedo* appeared to result more from decreased GS*albedo* rather than from nGS*albedo*. Previous studies reported that the feedback between snow/ice and *albedo* was due to the amplified warming on the Tibetan Plateau [2, 36, 43], which is similar to that at high northern latitudes [13, 45]. Annual *albedo* exhibited a decreasing trend during the study period, with snow/ice cover playing a weaker role than vegetation greenness in driving annual *albedo* on the plateau. This suggests that vegetation controls land surface *albedo* on the plateau, which is in agreement with other studies that have investigated high latitude ecosystems [8, 10, 15].

## Implications

Surface *albedo* is a critical variable in understanding the changes and regulations of energy balance in local and regional ecosystems, which alter climate at corresponding scales [3, 19, 46]. The magnitude of solar irradiance varies greatly between GS and nGS, and with elevation [2, 39, 40]. With an average regional base elevation that is greater than 4000 m and rapidly rising temperatures on the Tibetan Plateau, the lessons learned from this study will have profound implications in assessing and modeling the changes in ecosystem structure and function as a consequence of global climate change and local land use.

On the plateau, the effect of *albedo* on energy balance is amplified due to its high solar irradiance, which is greater than that in the arctic zone and in other low elevation inlands. At high latitudes (>50°N), solar radiation ranges between ~200 W m^−2^ in winter and 900 W m^−2^ in summer. For regions higher than 75°N, the lowest radiation value is zero and the highest value is ~600 W m^−2^ [47]. Across China, the average solar irradiance is ~170 W m^-2^ [48]. However, on the plateau, the solar irradiance is much higher than in the adjacent regions and has distinct seasonal dynamics, ranging from ~520 W m^−2^ in winter to 1000 W m^−2^ in summer [49]. Pistone *et al*. [50] reported that *albedo* in the Arctic region decreased from 0.52 to 0.48 between 1979 and 2011, which is equal to a 6.4 ± 0.9 W m^2^ addition of solar energy that has been absorbed by the Arctic Ocean region since 1979. These changes, combined with the predicted tundra-to-shrub land transition, caused the mean July air temperature to increase by 1.5–3°C in the Arctic [51]. Similarly, a greater value of solar irradiance and a more rapid change in vegetation greenness has been detected on the Tibetan Plateau than that in the tundra-to-shrub land transition, which would exacerbate the warming trend for the plateau and possibly East Asia.

Accurate modeling of the relationships between vegetation and *albedo* is essential for predicting the ecosystem’s overall response and feedback to a changing climate, especially in high altitude and latitude regions [8, 15, 52]. Favorable growing conditions are indicated by the lower surface *albedo* and higher radiation absorption abilities of well-developed vegetation [53–55]. However, anthropogenic disturbances (e.g., grazing) increase *albedo*. Quantitative separations of the positive/negative effects on *albedo* from these two driving forces at multiple scales are needed in future investigations.

An accurate estimation and prediction of the magnitude and spatiotemporal dynamics of *albedo* is crucial because of its potential feedbacks to regional climate and local change, such as reductions of permafrost, carbon emission, and melting snow/ice. At this point, however, it remains difficult to isolate the possible forces and feedback loops among land cover change (e.g., snow/ice cover, vegetation), *albedo*, surface temperature, etc. We expect accelerated changes on the Tibetan Plateau because of its low resistance to the changing climate and intensified land use [11]. More importantly, there are other processes affecting surface *albedo*, such as changes in lake size, urbanization, agricultural expansions, cloud patterns, etc. [56]. Another significant change in *albedo* may be derived from aerosol deposition on snow/ice, which has a great impact on reducing *albedo* globally [57]. Nevertheless, our results are consistent with the conceptualized reason that it is due to the significant decline in radiative convergence and surface sensible heat during the decades investigated [18]. These altered energy fluxes will produce direct and indirect effects on ecosystem processes. For example, if more solar radiation is absorbed, it will drain the soil and thereby alter soil moisture, biological microbial communities, nutrient cycling, etc. [2]. Consequently, plant growth will also suffer from increased water stress. Other related ecosystem processes would also change with altered soil condition, further resulting in possible limited plant growth and lower NDVI.

## Conclusions

Annual land surface *albedo* on the Tibetan Plateau has been decreasing since 2000. This decrease appeared to be caused mainly by lowered growing season *albedo*. A synchronous relationship was found for the change in the GSNDVI and GS*albedo* over time, as well as across the Tibetan landscape gradient. In the nGS, minimum *albedo* occurred in December—the coldest month of the year—which has the lowest average snow/ice cover. At high elevations (≥6000 m), WnGS*albedo*, non-GSSC, and soil moisture decreased during the study period. This study highlights not only the importance of monitoring ongoing NDVI and the effect of snow/ice on *albedo* closely, but more importantly, the change caused by vegetation on the Tibetan Plateau as well.

## Supporting information

S1 Data (RAR)
